# Microbiome in Eosinophilic Esophagitis—Metagenomic, Metatranscriptomic, and Metabolomic Changes: A Systematic Review

**DOI:** 10.3389/fphys.2021.731034

**Published:** 2021-09-10

**Authors:** Jordan D. Busing, Matthew Buendia, Yash Choksi, Girish Hiremath, Suman R. Das

**Affiliations:** ^1^Department of Pediatrics, Vanderbilt University Medical Center, Nashville, TN, United States; ^2^Division of Pediatric Gastroenterology, Hepatology, and Nutrition, Monroe Carrell Jr Vanderbilt Children's Hospital, Nashville, TN, United States; ^3^Department of Medicine, Vanderbilt University Medical Center, Nashville, TN, United States; ^4^Tennessee Valley Health System, Veterans Affairs, Nashville, TN, United States; ^5^Department of Otolaryngology and Head and Neck Surgery, Vanderbilt University Medical Center, Nashville, TN, United States

**Keywords:** eosinophilic esophagitis, metagenomics, metatranscriptomics, metabolomics, omics, metaproteomics, microbiome

## Abstract

**Background:** Our understanding of human gut microbiota has expanded in recent years with the introduction of high-throughput sequencing methods. These technologies allow for the study of metagenomic, metatranscriptomic, and metabolomic bacterial alterations as they relate to human disease. Work in this area has described the human gut microbiome in both healthy individuals and those with chronic gastrointestinal diseases, such as eosinophilic esophagitis (EoE).

**Objectives:** A systematic review of the current available literature on metagenomic, metatranscriptomic, and metabolomic changes in EoE was performed.

**Methods:** This review was performed following the PRISMA guidelines for reporting systematic reviews and meta-analyses. All relevant publications up to March 2021 were retrieved using the search engines PubMed, Google Scholar, and Web of Science. They were then extracted, assessed, and reviewed. Only original studies published in English were included.

**Results:** A total of 46 potential manuscripts were identified for review. Twelve met criteria for further review based on relevance screening and 9 met criteria for inclusion, including 6 studies describing the microbiome in EoE and 3 detailing metabolomic/tissue biochemistry alterations in EoE. No published studies examined metatranscriptomic changes. Samples for microbiome analysis were obtained via esophageal biopsy (*n* = 3), esophageal string test (*n* = 1), salivary sampling (*n* = 1), or stool specimen (*n* = 1). Samples analyzing tissue biochemistry were obtained via esophageal biopsy (*n* = 2) and blood plasma (*n* = 1). There were notable differences in how samples were collected and analyzed. Metabolomic and tissue biochemical alterations were described using Raman spectroscopy, which demonstrated distinct differences in the spectral intensities of glycogen, lipid, and protein content compared to controls. Finally, research in proteomics identified an increase in the pro-fibrotic protein thrombospondin-1 in patients with EoE compared with controls.

**Conclusions:** While there are notable changes in the microbiome, these differ with the collection technique and method of analysis utilized. Techniques characterizing metabolomics and tissue biochemistry are now being utilized to further study patients with EoE. The lack of published data related to the human microbiome, metagenome, metatranscriptome, and metabolome in patients with EoE highlights the need for further research in these areas.

## Introduction

Eosinophilic esophagitis is a chronic, food, and/or aeroallergen-mediated inflammatory disease (Liacouras et al., 2011; Wechsler and Bryce, 2014; Davis and Rothenberg, 2016). It is characterized clinically by symptoms of esophageal dysfunction such as vomiting, abdominal pain, and dysphagia. The diagnosis is confirmed by the presence of intense eosinophilic inflammation (> 15 eosinophils per high-power field [eos/hpf]) in esophageal mucosal biopsies (Liacouras et al., 2011). The prevalence of EoE is estimated to be 1 in 2000 in the United States. The disease adversely affects the quality of life of patients and imposes a substantial financial burden on the healthcare system (Jensen et al., 2015; Jensen and Dellon, 2018).

The current disease paradigm is that a combination of genetic predisposition, dysregulated immunity, and environmental factors contribute toward the development of EoE (Lehman and Lam, 2019). While substantial progress has been made in understanding the role of genetics and immune response, there is growing interest on the impact of environmental factors on the development and progression of EoE. Among environmental factors, early studies on the microbiome in EoE have focused on characterizing which microbes are more common in disease, but more recent studies have investigated the function of those microbes, how they interact with the host machinery, and which substrates are transformed during cellular and biochemical metabolism.

In recent years, advancements and increased access to high-throughput sequencing technologies have expanded our understanding of the role of the human microbiome in various disease states. These approaches have also facilitated metagenomic and metatranscriptomic investigations of the interactions between host tissues and their microbial communities. Metagenomics is the study of the collective genetic material of the human microbiome (Bikel et al., 2015). This process maps genes to characterize the putative functional pathways which allows insight into the abundance and genetic potential within the microbial community present. Metagenomics, while powerful, doesn't discriminate between live and dead bacteria. A sample can still possess DNA from bacteria regardless of whether that bacteria is currently living or not. Metagenomics is limited to sequencing DNA that is merely present, but that does not necessarily provide insight into which bacterial are alive and active in the sample. Metatranscriptomics describes whole-genome analysis and mapping of the expressed pathways, which allows for determination of which microorganisms are actively involved in the disease phenotype. Similarly, metabolomics is the study of small molecules produced by cells (Patti et al., 2012). The presence and alterations of microbial metabolites such as lipids, carbohydrates, amino acids can provide direct insight into biochemical alterations which lead to phenotypic presentation of disease. The use of these technologies allows for the expansion of the central dogma of molecular biology—DNA(genomics) to RNA(transcriptomics) to protein (proteomics) to metabolite (metabolomics)—to better understand the epigenetic and post-translation modification (Patti et al., 2012). Thus, metagenomic sequencing, along with metatranscriptomics and metabolomics (multi-omics), can help characterize the functional relevance of bacterial gene expression, while also potentially providing insight into the mechanistic role of the microbiome in EoE. In this state-of-the art review, we aim to summarize what is known about the microbiome, metagenomics, metatranscriptomics, and metabolomics in EoE.

## Methods

Our methods adhere to the guidelines established by Preferred Reporting Items for Systematic Review and Meta-Analyses (PRISMA) (Page et al., 2021). To identify relevant studies, we conducted a search in PubMed, Google Scholar, and Web of Science. To optimize the search, no date limits were imposed. The last search was performed on March 1st, 2021. A combination of search terms such eosinophilic esophagitis/oesophagitis (EoE), microbiome (or microbiota), human microbiome (or microbiota), genome (or genomics), metagenomics (or metagenome), transcriptome (or transcriptomics), metatranscriptome (or metatranscriptomics), or metabolome (or metabolomics) were used.

We included publications which detailed original data and descriptions of the terms mentioned above. To ensure quality, non-original articles, non-human studies, and abstract-only publications were excluded. Additionally, studies published in languages other than English were also excluded.

Two authors (M.B., J.B.) evaluated articles for eligibility and quality. Each person extracted data independently. Variables that were sought were obtained using a standard form that was designed to collect title, author, publication year, country of origin, study type, specific aims, research methods, and conclusions. Risk of bias was assessed independently by two investigators (M.B., J.B.) Any disagreement between authors in data abstraction or bias was resolved by discussion with the senior authors (S.D. and G.H.) and review of the publications. This approach allowed for the minimization of bias.

## Results

**Figure 1:**
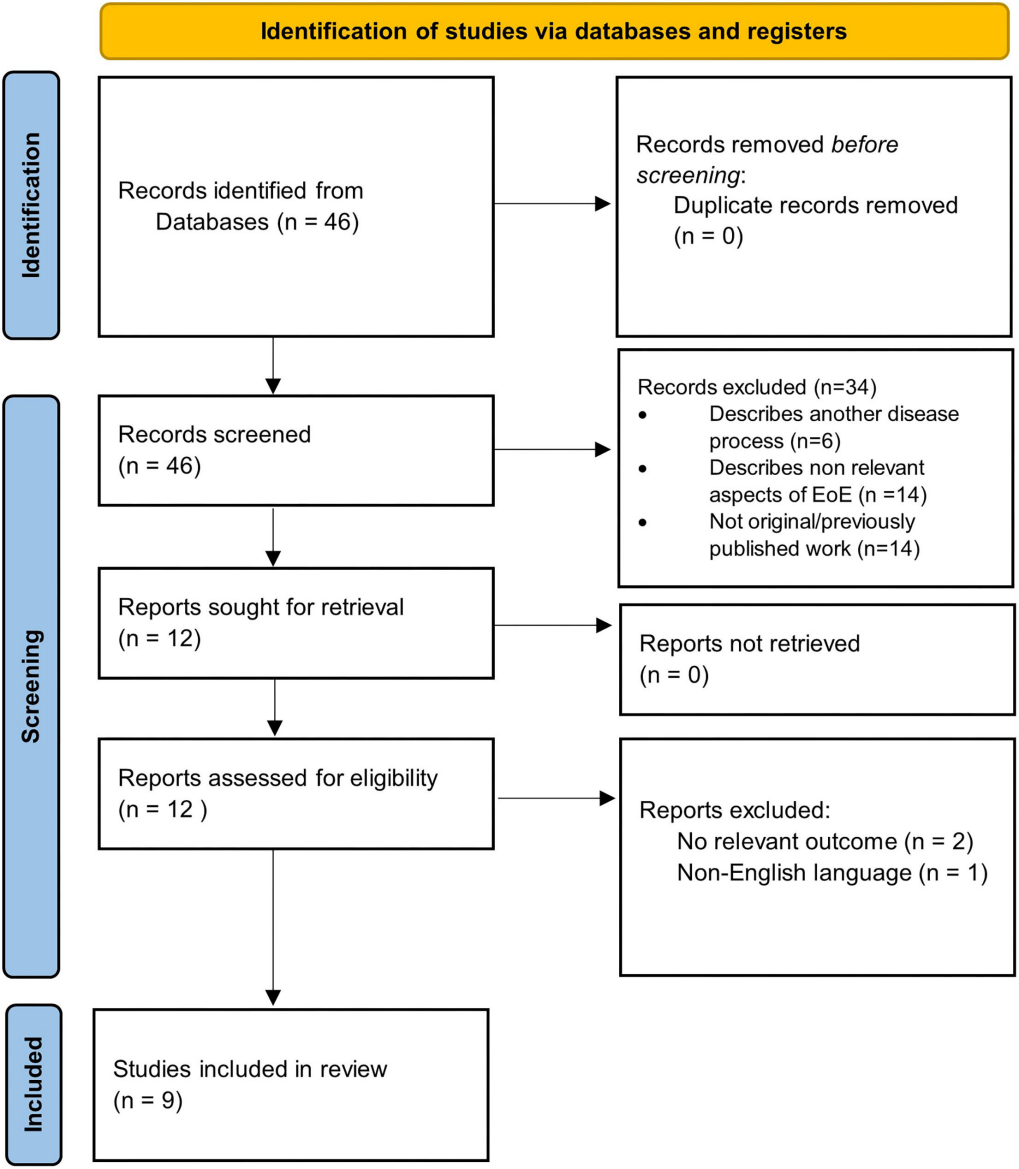
PRISMA 2020 flow diagram detailing selection process of included studies.

**Figure 2:**
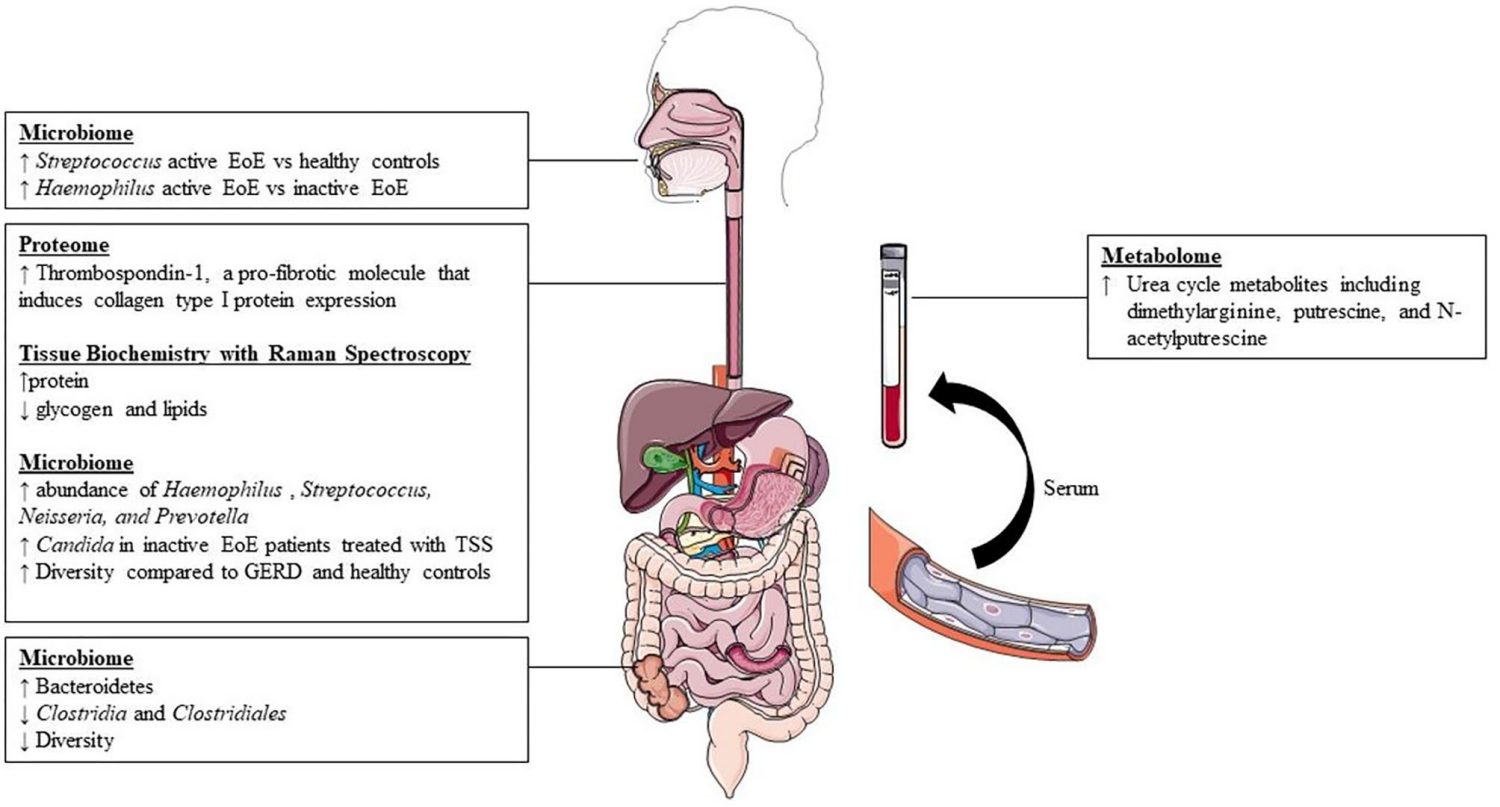
Summary of available findings from literature.

Our search yielded a total of 46 studies, all of which were reviewed by abstract and title. There were 34 articles excluded following initial screening. Articles were excluded following screening because they described another disease process (*n* = 6), were not original data/were systematic reviews (*n* = 14), or described non-relevant aspects of EoE (*n* = 14). After the initial screening process, a total of 12 articles were sought for retrieval. Publications that were not in English language or had no relevant outcomes were excluded, leaving a total list of 9 articles for inclusion (Figure 1). The results of the studies are summarized in Figure 2. Each study is outlined in Table 1.

**Table 1:** Summary of studies applying microbiome analysis in eosinophilic esophagitis.

**Study**	**Study population**	**Methods**	**Findings**
**Microbiome**			
Benitez et al. (2015)	• Sample size: *n* = 68 • Oral swabs and esophageal biopsies from 33 EoE patients and 35 controls	• 16S rRNA gene sequencing (V1 to V2)	• Higher abundance: *Streptococcus, Neisseria*, and *Prevotella*
Benitez et al. (2021)	• Sample size: *n* = 79 • Esophageal biopsies from 69 EoE patients (36 inactive, 33 active) and 10 controls	• 16S rRNA gene sequencing (V1 to V2) and internal transcribed spacer (ITS) sequencing	• EoE patients: decreased abundance of *Alloprevatella* • Increase in the abundance of *Haemophilus* to inactive to active EoE. • Increase in *Candida* in inactive EoE patients treated with TSS when compared with those not treated with TSS.
Harris et al. (2015)	• Sample size: *n* = 70 • Esophageal string test from 37 children with EoE, 8 with GERD, and 25 controls	• 16S rRNA gene sequencing (V1 to V2)	• Significant increased *Haemophilus* in patients with EoE that returns to controls when disease is controlled.
Hiremath et al. (2019)	• Sample size: *n* = 35 • Saliva samples from 26 children with EoE and 19 controls	• 16S rRNA gene sequencing (V4 region)	• Higher abundance: *Streptococcus* was more abundant with active EoE vs. non-EoE controls, *Haemophilus* was more abundant in active EoE vs. inactive EoE • Diversity: no significant difference
Norder Grusell et al. (2018)	• Sample size: *n =* 27 • Oral punch biopsies and brush samplings from the oral cavity (as well as brush samplings and endoscopy biopsies of upper and lower esophagus) of 17 patients with GERD and 10 with EoE	• Culture	• Higher abundance: *Streptococcus* (viridians) was the most common bacteria in both groups • Diversity: decreased in GERD vs. EoE
Kashyap et al. (2019)	• Sample size: *n =* 20 • Stool bacterial DNA from 12 patients with EoE and 12 controls	• 16S rRNA gene sequencing (V4 region)	• Significant decreases in *Clostridia* and *Clostridiales* in patients with EoE. • Diversity: decreased in EoE
**Proteomics and tissue biochemistry**			
Hsieh et al. (2021)	• Sample size *n =* 10 • Esophageal biopsy from 5 healthy donors and 5 with therapy refractory EoE.	• Esophageal biopsy with fibroblasts placed on autologous or non-autologous decellularized ECM	• Increased hrombospondin-1, a pro-fibrotic molecule that induces collagen type I protein expression, is increased in patients with EoE compared to controls.
Hiremath et al. (2020)	• Sample size *n =* 24 • Children with active EoE (*n =* 8) and inactive EoE (*n =* 6) and non-EoE controls (*n* = 10)	• Utilized Raman Spectroscopy to profile and compare esophageal samples	• Raman peaks attributable to glycogen content was lower in children with active EoE compared with that in non-EoE controls • Protein intensity was higher in children with aEoE compared with that in non-EoE controls. • Raman peaks attributable to glycogen and lipid inversely correlated with eosinophilic inflammation and basal zone hyperplasia.
**Metabolomics**			
Moye et al. (2018)	• Sample size *n =* 24 • Children with EoE (*n =* 7) and children on PPI (*n =* 11, 4 with EoE) comparted to healthy controls not on PPI (*n =* 6)	• Blood sample profiling using the subclasses: amino acids, tricarboxylic acid cycle, acetylation, and methylation. 48 metabolites measured in total.	• Increased urea cycle metabolites including dimethylarginine, putrescine, and N-acetylputrescine in patients with EoE

### Microbiome, and Eosinophilic Esophagitis

Currently, culture-independent sequencing utilizing amplicon-based 16S ribosomal RNA (a highly conserved region amongst bacteria) is the most common form of molecular sequencing (Li et al., 2018). Bacterial 16S ribosomal RNA (rRNA) genes generally contain nine “hypervariable regions” (V1-V9) that are used for species identification (Chakravorty et al., 2007). While 16S rRNA sequencing has limitations, this method utilizing hypervariable regions V1-V9 is a cost-effective, efficient, and unbiased method which captures the microbial community structure and composition and has been utilized in most of the previous work done examining the esophageal, oral, and stool microbiome in patients with EoE. Using oral and esophageal swabs and subsequent 16S sequencing with V1 to V2 primers, Benitez et al. showed that patients with active EoE have a distinct esophageal microbiome as compared with non-EoE controls. This study included 68 subjects ages 2–18 years old. They demonstrated that the normal esophageal microbiome is dominated by *Firmicutes* species. However, when compared with non-EoE controls, patients with active EoE had increased abundance of *Neisseria* and *Corynebacterium*, both Proteobacteria. Interestingly, patients with inactive EoE did not have significant differences compared to healthy controls. Additionally, they showed that enrichment of the bacterial genuses *Granulicatella* and *Campylobacter* occurred with re-introduction of highly allergenic foods (dairy, wheat, nuts, eggs, soy, and shellfish) to both the active and inactive EoE cohorts' diet (Benitez et al., 2015). This group also examined the oral and esophgeal microbiome to determine if the oral microbiome was like that of the esophageal microbiome, and thus could serve as a less invasive surrogate for study of the esophageal microbiome. They show a modest, but significant correlation between these two environments. This correlation was unaffected by disease status. They conclude, however, that the oral microbiome was unchanged in patients with active, inactive, and healthy controls and thus their data did not recommend using oral samples in place of esophageal samples for disease monitoring (Benitez et al., 2015).

In a different study, Harris et al. used the esophageal string test to sample esophageal luminal secretions and then performed subsequent 16s rRNA sequencing using V1 to V2 primers to study the esophageal microbiome of patients with EoE and GERD compared to healthy controls (Harris et al., 2015). This study included both children and adults and the majority of patients were Caucasian males. They found a significant increase in the relative abundance of *Haemophilus* in patients with untreated EoE compared to healthy controls. The abundance of *Haemophilus* was reduced to levels similar to that in controls and patients with GERD when the histologic remission of EoE was achieved through either swallowed steroids or dietary measures. This highlighted that treatment can play a role in altering the microbial community of the esophagus in patients with EoE.

In a follow-up study to their 2015 study, Benitez et al. used both 16S rRNA gene and internal transcribed spacer (ITS) sequencing to examine the effect of topical swallowed corticosteroids on esophageal bacterial and fungal populations in EoE (Benitez et al., 2021). This study included 69 children with EoE, 33 with active EoE and 36 with inactive EOE. There were 10 healthy controls. They found that *Streptococcus, Prevotella*, and *Alloprevatella* dominated the esophageal microbiota in all studied patients, including controls. Both active and inactive EoE patients had decreased abundance of *Alloprevatella* when compared to non-EoE controls. There was a stepwise increase in the abundance of *Haemophilus* from control to inactive to active EoE. This is also the first group to examine the fungal component of the microbiome present in EoE, including patients being treated with topical swallowed steroids. *Candida, Cladosporiaceae*, and *Malassezia* were the most common fungal taxa in all groups. Other important findings included an increased proportion of *Candida* in non-EoE controls compared to steroid-naïve EoE subjects and changes in the fungal community following treatment with topical swallowed steroids—namely, significant increase in *Candida* in inactive EoE patients treated with topical swallowed steroids (TSS) when compared with those not treated with TSS (Benitez et al., 2021).

In 2018, Grussell et al. utilized traditional culture driven results of brush samplings and mucosal punch biopsies from the oral cavity and esophagus instead of molecular sequencing to compare microbiota in adult patients with GERD and EoE to healthy controls (Norder Grusell et al., 2018). This group found alfa-streptococci was the most common group in all three patient populations and patients with EoE had significantly more diversity compared to healthy controls. Additionally, they confirm findings by Harris et al. and Benitez et al. with increased abundance of *Haempholius* in patients with EoE compared to individuals with GERD and healthy controls (Norder Grusell et al., 2018).

More recently, Hiremath et al. used 16s rRNA sequencing utilizing the V4 region to characterize the salivary microbiome in children with EoE (Hiremath et al., 2019). They found *Streptococcus* was more abundant with active EoE vs. non-EoE controls. *Haemophilus* was more abundant in active EoE vs. inactive EoE and positively correlated with esophageal endoscopic and histologic disease activity. This increase in *Haemophilus* with active EoE mirrors previous findings (Harris et al., 2015). They also note a trend toward lower microbial richness and alpha diversity in children with EoE (Hiremath et al., 2019).

Finally, Kashyap et al. studied the stool microbiome in patients with EoE (Kashyap et al., 2019). This group used 16s rRNA sequencing utilizing the V4 region to compare the stool microbiota of 12 patients with EoE to 12 healthy controls. This group found significant decreases in *Clostridia* and *Clostridiales* in patients with EoE. They also note decreased stool microbial diversity in patients with EoE compared to controls.

### Transcriptomics, Metatranscriptomics, and Eosinophilic Esophagitis

Studies describing the EoE transcriptome have allowed researchers to further understand disease mechanisms of EoE. They are discussed here in brief to highlight how the transcriptome has contributed to the understanding of EoE and further display how further understanding of the metatranscriptome could be important. Blanchard et al. first utilized whole-genome wide transcript oligonucleotide-based DNA microarray chips with esophageal biopsies to define the EoE transcriptome (Blanchard et al., 2006). They subsequently identified 574 transcripts—colloquially known as the EoE transcriptome—that were expressed differently in children ages 2–17 with EoE compared to healthy controls. These altered genes have varying function and play a role in several areas including immunity, inflammatory response, barrier function, atopy, and eosinophilia (Sherrill et al., 2014). Wen et al. expanded further on this work and developed an EoE molecular diagnostic panel by utilizing quantitative PCR on fixed paraffin embedded esophageal biopsy samples (Wen et al., 2019). This panel utilized 96 genes to accurately identify EoE in adults and children with 96% sensitivity and 98% specificity. Importantly, this the EoE diagnostic panel was able to differentiate active EoE vs. controls, EoE in topical steroid remission vs. controls, and EoE vs. GERD (Wen et al., 2013).

Genes involved in epithelial barrier dysfunction and T helper type-2 mediated immune dysregulation are thought to be central to the pathogenesis of EoE (Lyles and Rothenberg, 2019). Current genes implicated in epithelial barrier dysfunction include CAPN14, DSG1, FLG, and SPINK5, and SPINK7. Additionally, CCL26, involved in eosinophil chemotaxis, and TSLP, involved in dendritic cell chemotaxis, have been implicated in T helper type-2 mediated immune dysregulation (Lyles and Rothenberg, 2019).

More recent work by Wen et al. has focused on single cell RNA sequencing to investigate resident esophageal T-cells in patients with EoE compared to healthy individuals and those with EoE in remission (Wen et al., 2019). Important findings include identification of eight T-cell subclasses that are increased in active EoE inflammation, including the two most highly upregulated populations: Treg cells (FOXP3+) and effector Th2-like (GATA3+) cells.

While much work has been completed to describe transcriptomic changes in EoE, briefly highlighted above, little has been done to characterize the metatranscriptome. Specifically, the expression profile of microorganisms which are altering host biology has been understudied and is necessary before metatranscriptomic analysis can be done. Metatranscriptomic data is a powerful tool that would improve our understanding of how previously identified microbiota in active EoE might alter the functional profile of the esophageal epithelial gene expression.

### Metabolomics and Tissue Biochemistry

Our understanding of the pathogenesis of EoE remains incomplete due to limitations in characterizing not only the microbiome, but also the biomolecular and biochemical alterations that are present in the esophageal epithelium of these patients (Hiremath et al., 2020). Several groups have recently described the protein expression pattern in patients with EoE.

Hiremath et al. utilized Raman spectroscopy and proteomic analysis of esophageal mucosa to demonstrate that patients with EoE had distinct differences in spectral intensities of glycogen, lipid, and protein content compared to controls (Hiremath et al., 2020). The findings were able to use Raman spectroscopy to reliably distinguish between controls, active EoE, and inactive EoE. Specifically, peak intensity for glycogen was decreased and peak intensity for proteins increased for patients with active EoE compared to healthy controls. Additionally, peak intensity for lipids was higher in children with inactive EoE compared to those with active disease. This group hypothesized that a decrease in glycogen and lipids in the epithelium of patients with active disease could be due to increased uptake of glycogen by eosinophils and increased amounts of undifferentiated epithelial cells (i.e., basal cell hyperplasia) with decreased cytoplasmic glycogen volume. They postulate that lipid content could be higher in children with inactive EoE because of the underlying mucosal healing process. Finally, they hypothesized that increased protein content in EoE could be related to increased chemokines and cytokines mediating inflammation.

Hsieh et al. recently described unique changes to the extracellular matrix proteome in patients with EoE (Hsieh et al., 2021). This group isolated fibroblasts from 5 children with active EoE and 5 healthy controls and utilized extracellular matrix (ECM) from both groups to culture these fibroblasts. The goal of this study was to determine how the extracellular matrix of patients with EoE can alter the function of normal fibroblasts. Fibroblasts from healthy controls that were cultured on ECM from patients with active EoE demonstrated higher levels of type 1 collagen and α-smooth muscle actin when compared to control fibroblasts cultured on autologous ECM. The authors then analyzed both sets of ECM and subsequently demonstrated that thrombospondin-1, a pro-fibrotic molecule that induces collagen type I protein expression, is increased in patients with EoE compared to controls. This work highlights how further insight into proteomics can further our understanding of disease specific long-term sequelae.

Work has also been completed to not only catalog which proteins are differentially expressed in EoE, but also to describe whether there are differences in measurable metabolites. In 2019, Moye et al. was the first group to describe key blood plasma metabolite changes in children with EoE compared to healthy controls (Moye et al., 2018). They found differences in metabolites between healthy controls and patients with EoE. Notably, they identified key differences in patients with EoE both on and off proton pump inhibitor therapy. Based on their work, they suggest dimethylarginine, putrescine, and N-acetylputrescine as potential biomarkers for EoE.

## Discussion

In this review, we summarize the published data related to human microbiome and EoE and highlight what is known about the metagenome, metatranscriptome, and metabolome. Our major conclusion is that there is significantly more work needed in these areas to characterize the complex role of the gut microbiome in EoE. At this time, transcriptomics (tissue microarray, bulk RNA sequencing, and single cell RNA sequencing) has been done, but independent of the gut microbiome. There is no published work to our knowledge integrating the immune response, gene expression, and microbiome. Similarly, little work has been done regarding metabolomics and metaproteomics—advances in these fields could give insight into potential non-invasive disease monitoring or therapeutic targets.

The goal of the study of the human microbiome is to characterize the microbial community, its interaction with the host, and its role in human health and disease. To date, 16S RNA sequencing is the most common method for studying the human microbiome. This method utilizes highly conserved bacterial regions to identify bacterial RNA within a sample and hypervariable region to identify and quantify different species within a sample (Bikel et al., 2015). This method is utilized for two primary reasons—it is fast and cost effective. However, 16S RNA sequencing is not without its limitations in characterizing the microbiota in a sample (Li et al., 2018). First, adequate sequencing is limited by primer selection, which can alter the apparent abundance of specific communities within a given sample (Bikel et al., 2015). Second, external factors such as reagent contamination and varying amplification cycling conditions can affect results (Salter et al., 2014; Eisenhofer et al., 2019; Stinson et al., 2019). Third, 16S sequencing is limited to using only known primers resulting in the potential for excluding unknown sequences and thus excluding bacterial species present in a sample (Ross et al., 2012).

While these initial studies have provided important preliminary data, they demonstrate the limitations of 16S rRNA sequencing in patients with EoE and highlight the varying sampling methods used for analysis. Given the complexity of the host microbiome, differences in sampling and analysis lead to further difficulties deriving meaningful information from these studies. In the six studies above, there were various methods utilized to characterize the EoE microbiome. First, both culture independent with 16S rRNA sequencing and traditional culture driven methods were utilized. These methods can produce different results from the same sample. Not all bacteria can be cultured using traditional media, leading to exclusion of bacterial species. An advantage of culture driven data vs. culture independent approaches is that advanced sequencing techniques utilizing rRNA can potentially amplify dead bacteria and skew results toward bacteria that are not actually active in a sample (Norder Grusell et al., 2018). Theoretically, this could amplify bacteria that play no active role in the disease process but are identified because of the sequencing method selected. Second, in studies that used 16s rRNA, two groups used primers from the V4 region while three groups used primers V1-V2. This difference can lead to skewed identification based on primer selection; however, there is no consensus on which hypervariable region is most appropriate for use in EoE. Different primers are selected for a variety of reasons, including cost and lab availability. Finally, the sampling sites and methods of these studies varied. These include traditional esophageal biopsy, esophageal string test, saliva sampling, and stool collection. There is no consensus on the most accurate way to collect microbial data from esophageal tissue, but esophageal biopsy obtained via endoscopy has traditionally been the gold standard. It is possible that different sampling techniques utilized could play a role in varying results outlined above. For example, the researchers that utilize the string test for sampling provide strong evidence that this test accurately captures esophageal microbial composition when compared with biopsy results.

While 16S sequencing is a valuable tool for quantifying the composition and abundance of bacteria in a sample, it does little to describe the function of individual bacteria within a microbial community. The full genetic make-up and function of a microbial community can be described better through the study of metagenomics, metatranscriptomics, and metabolomics. Metagenomics characterizes the gene content and functional potential of a microbial sample. Metatranscriptomics describes the functional genetic profile of a particular sample at a given point in time by characterizing the mRNA expression of the microorganisms in the sample. In other words, as Li et al. state, metagenomics and metatranscriptomics characterize a bacterial community by identifying “who they are” and what they do” (Li et al., 2018).

Metabolomics is the study of the down-stream effect of this genetic material that identifies the metabolites produced by a microbial community. Metabolomics describes a high-throughput technique for directly measuring biochemical activity by monitoring the substrates and biochemical products produced during cellular metabolism (Patti et al., 2012). Taken together, these techniques can fully characterize a microbial community by describing the microbial species present, ascertaining differentially expressed transcripts, and defining the metabolites produced as a result of altered gene expression.

These advances in sequencing techniques are not without limitations. Human DNA can interfere with the ability to correctly sample the microbiome and is highly dependent on the site from which the sample is taken (Bikel et al., 2015). This leads to potential for large sequences of DNA to be eliminated because they are derived from the host instead of the microbial community, which is wasteful and can be cost prohibitive (Bikel et al., 2015). Outside of cost, other relevant challenges also include lack of adequate reference databases, inability to differentiate active vs. inactive members, and sequencing such a large array of microorganisms (Shakya et al., 2019).

One challenge in the use of advanced sequencing techniques is interpreting the large volumes of data produced and attempting to determine how that data can be used to advance real life implications of disease. While the amount of information derived from study of the -omics can seem daunting, others have shown how integration of these techniques can directly lead to important discoveries. For instance, van Dam et al. gained valuable insight into gene expression of *M. tuberculosis* by utilizing omics co-expression networks that would have not been possible by focusing on only one type of analysis in isolation (Van Dam et al., 2014).

While there has been work done to characterize the microbiome in patients with EoE, little work has been done in these fields. Study of advanced genomics in EoE represents a substantial knowledge gap which needs to be filled in the future to advance the characterization and treatment of EoE.

## Data Availability Statement

The original contributions presented in the study are included in the article/supplementary material, further inquiries can be directed to the corresponding author/s.

## Author Contributions

MB and JB contributed toward literature search, identifying relevant publications, data extraction, interpreting the results, drafted the manuscript, and designed the tables and figures. SRD, GH, and YC contributed toward conceiving and designing the study, literature search, identifying relevant publications, data extraction, interpreting the results, drafted the manuscript, and designing the tables and figures. All authors contributed to the article and approved the submitted version.

## Funding

GH is supported by the American College of Gastroenterology Junior Faculty Development Award, and the Eunice Kennedy Shriver National Institute of Child Health & Human Development of the National Institutes of Health (NIH) under the Award Number K12HD087023. SRD is currently supported by NIAID (R21AI154016-01, R21AI149262, R21AI14232, R21AI142321-01A1S1, U19AI095227, and P30AI110527), NHLBI (1R01HL146401), CDC (75D3012110094), and the start-up funds from Vanderbilt University Medical Center awarded to SRD.

## Conflict of Interest

The authors declare that the research was conducted in the absence of any commercial or financial relationships that could be construed as a potential conflict of interest.

## Publisher's Note

All claims expressed in this article are solely those of the authors and do not necessarily represent those of their affiliated organizations, or those of the publisher, the editors and the reviewers. Any product that may be evaluated in this article, or claim that may be made by its manufacturer, is not guaranteed or endorsed by the publisher.
